# Cross-Subject Emotion Recognition Using Fused Entropy Features of EEG

**DOI:** 10.3390/e24091281

**Published:** 2022-09-11

**Authors:** Xin Zuo, Chi Zhang, Timo Hämäläinen, Hanbing Gao, Yu Fu, Fengyu Cong

**Affiliations:** 1 School of Biomedical Engineering, Faculty of Electronic Information and Electrical Engineering, Dalian University of Technology, Dalian 116024, China; 2 Faculty of Information Technology, University of Jyväskylä, 40014 Jyväskylä, Finland; 3 Liaoning Key Laboratory of Integrated Circuit and Biomedical Electronic System, Dalian 116024, China

**Keywords:** emotion recognition, EEG, feature fusion, MSE, BiLSTM

## Abstract

Emotion recognition based on electroencephalography (EEG) has attracted high interest in fields such as health care, user experience evaluation, and human–computer interaction (HCI), as it plays an important role in human daily life. Although various approaches have been proposed to detect emotion states in previous studies, there is still a need to further study the dynamic changes of EEG in different emotions to detect emotion states accurately. Entropy-based features have been proved to be effective in mining the complexity information in EEG in many areas. However, different entropy features vary in revealing the implicit information of EEG. To improve system reliability, in this paper, we propose a framework for EEG-based cross-subject emotion recognition using fused entropy features and a Bidirectional Long Short-term Memory (BiLSTM) network. Features including approximate entropy (AE), fuzzy entropy (FE), Rényi entropy (RE), differential entropy (DE), and multi-scale entropy (MSE) are first calculated to study dynamic emotional information. Then, we train a BiLSTM classifier with the inputs of entropy features to identify different emotions. Our results show that MSE of EEG is more efficient than other single-entropy features in recognizing emotions. The performance of BiLSTM is further improved with an accuracy of 70.05% using fused entropy features compared with that of single-type feature.

## 1. Introduction

Emotion is a specific psychological and physiological response generated by perceiving external and inner stimuli. It is a complex state combining thoughts, feelings, and behaviors and is an important part of daily human life [1]. Previous studies have demonstrated that emotion plays a vital role not only in the process of perception, decision making, and communication but also in the learning and memory process [2]. As a result, the measurement and characterization of different emotion states are of great importance to emotion recognition-related studies both theoretically and practically. For example, emotion recognition can be widely used in areas such as health care, distance learning, and user experience evaluation of products, which are closely related to humans [3]. Furthermore, it contributes to the computer ability of emotion recognition and expression in the human–computer interaction (HCI) field [4]. As emotion is often accompanied by high cognitive activities of the brain involving complex psychology and physiology processes [5], further study on how to recognize different emotions accurately is necessary.

There are many kinds of approaches to recognizing emotion states in existing studies. According to the data used in emotion recognition, they can be roughly divided into two categories. One category is based on non-physiological signals, whereas the other is based on physiological signals. Conventional emotion recognition methods based on non-physiological signals usually use facial expressions, behaviors, and voice-based signals, etc.. The features of these signals are more obvious for observation and easier to be extracted. Jain et al. proposed deep convolutional neural networks for observing emotion states based on different facial motions in different image emotions [6]. Meng et al. developed a speech emotion recognition method using spectrum features of speech signals [7]. There is also emotion recognition research combing different types of non-physiological signals. For instance, Kessous et al. studied a multimodal automatic emotion recognition method using the Bayesian classifier based on a mixture of facial expressions, gestures, and acoustic signals, and they found that fusing the multimodal signals would largely increase classification accuracy compared with unimodal systems [8]. Although the data collection process of these methods is easier, their availability and reliability could not be completely guaranteed, as they are mainly affected by two factors [9]. On the one hand, effective non-physiological signals are hard to obtain from participants who have trouble expressing their feelings through body language. On the other hand, participants can deliberately control their expressions, tone, and postures to hide their real feelings. Contrary to the non-physiological measurements, physiological signals are more reliable and effective, as these signals originate from spontaneous activities of the nervous system which cannot be controlled intentionally [1]. The mostly frequently used physiological signals include autonomic nervous system (ANS) signals such as the electrocardiogram (ECG), the electromyogram (EMG), skin resistance, and blood pressure, and neutral nervous system signals such as EEG, functional magnetic resonance imaging (fMRI), and so on. Kim et al. analyzed the multimodal autonomic physiological signals (i.e., ECG, EMG, respiration, and skin conductivity) induced by music and developed a scheme of emotion-specific classification [10]. The brain signals obtained directly from the neutral nervous system can reflect the dynamic neuro-electrical changes in real-time with high resolution, compared to ANS signals which often include a time delay [9]. In addition, the activity of EEG signals varies in different brain regions while emotional processes occur. Particularly, the lateral temporal brain areas are more active than other areas, and the energy of EEG increases for positive emotion, whereas lower energy appears in neutral and negative emotions [11]. Therefore, the emotional changes in different emotion states can be measured by EEG signals in the lateral temporal region. What is more, the equipment of EEG collection is small in size, portable, and much cheaper than that of fMRI. EEG-based emotion recognition has become one of the most prosperous research fields.

To recognize emotion states accurately based on EEG signals, features revealing the dynamic changes of EEG under different emotions should first be extracted. There are four main types of features used in EEG-based emotion recognition [12]: time domain features (e.g., statistical features and auto-regression coefficient), frequency domain features (e.g., power spectral density and energy spectrum), time-frequency features (e.g., wavelet coefficients), and non-linear dynamic features (e.g., fractal dimension and entropy features). The ability of different features varies in reflecting emotion states. Energy-based features in different brain regions have been commonly adopted in emotion recognition. As different brain regions are activated in different emotions, the energy of different frequency bands in these brain regions can be used for emotion recognition [13]. Du et al. selected sound clips of three affective states (i.e., happy (high arousal), afraid (high arousal), and neutral (low arousal)) to explore frontal asymmetry [14]. Their research demonstrated that the right frontal region is more related to high-arousal emotions (i.e., happiness and fear), whereas the left frontal region correlates with low-arousal emotions (i.e., neutral); thus, the energy asymmetry between the left and right brain can be used to classify emotion states. Further, Liu et al. found that there is a correlation between the emotional states and EEG frequency bands and that high-frequency bands contain more emotional information than low-frequency bands [15]. EEG signals, which are a direct reflection of brain activities, are non-stationary signals with a low signal-to-noise ratio, and the activation of EEG and the information it contains varies in different emotions. It is difficult to analyze EEG signals using only traditional time- or frequency-domain features. In recent years, entropy-based features have been proven to manifest more complex dynamic information in EEG than conventional features, leading to a wide use in many fields [16]. Wang et al. extracted the sample entropy (SE) feature of overnight sleeping EEG data utilizing the assisted sliding box algorithm to show the dynamic changes and a reduction of computation time [17]. Chen et al. proposed a method using the approximate entropy (AE) feature and its transformation to identify four human emotions based on EEG with an accuracy of 83.34% [18]. Zheng et al. trained a Deep Belief Network (DBN) to classify three emotions with the input of the differential entropy (DE) feature [19]. Their results showed that the DE feature of certain brain regions could reflect the dynamic changes of EEG in different emotions and can be used for recognizing emotion states. What is more, entropy features deployed to analyze brain states in other areas may also contribute to emotion recognition. For instance, multiscale entropy (MSE), calculating entropy in multiple time scales, has been proven to achieve better robustness of results than conventional features in fields of disease diagnosis and sleep studies [20]. Hadoush et al. adopted MSE to explore patterns in children with mild and severe autism spectrum disorders (ASD) and found that MSE could serve as an effective index for the severity of ASD [21]. Vladimir et al. explored the changes in brain signal complexity across several distinct global states of consciousness using MSE [22]. The results indicated that MSE changes throughout the sleep cycle and is strongly time-scale dependent, which makes it possible to use MSE for sleep staging. However, a challenge still exists in analyzing EEG signals based on entropy features. These different features characterize the EEG implicit complexity information to varying degrees, but it remains unclear which type of entropy feature is more effective for describing emotional states. Further, previous studies demonstrated that any feature could add complementary information to the other features [23]. Hence, there is a necessity to integrate the advantages of different entropy features to enhance the performance of emotion classification.

To take advantage of the EEG features, researchers have trained a variety of classifiers to recognize different types of emotion states. Traditional classifiers such as SVM, K-Nearest Neighbor (KNN), and transfer learning are widely used for emotion classification. Liu et al. established a real-time EEG-based emotion recognition system using SVM that could successfully classify positive and negative emotions with acceptable results [24]. Kolodyazhniy et al. extracted features from physiological signals induced by different emotional film clips and proposed an affective computing approach based on the KNN classifier [25]. Lan et al. [26] utilized the DE feature of EEG and the transfer learning technique to detect three emotions reaching an accuracy of 72.47% in the SEED dataset. Although traditional classifiers have achieved different recognition performance in simple tasks (e.g., 86.43% accuracy for three positive emotions in [24], 83.34% accuracy for four different emotions in [18], 77.5% accuracy via different types of signals in [25]), they are not efficient enough to learn the contextual dependency in a time series and do not perform well in cross-subject emotion recognition [27]. As we all know, human emotions are a continuous time series, and the current emotion state is influenced by both the current stimulus and previous emotions. In this case, it is difficult for traditional classifiers to recognize human emotion only based on the current feature. The Bidirectional Long Short-term Memory (BiLSTM) network has the ability to learn long- and short-term dependency between time steps and to memorize both forward and backward contextual information in a time series, compared with the one-directional Long Short-term Memory (LSTM) network [28] that is widely used in speech synthesis, pathological voice detection, and motion prediction [29,30,31]. It has been proven to perform effectively in pattern recognition and has been successfully deployed in sequence-to-sequence classification tasks in many fields [32,33,34,35]. Mahmud et al. trained an automated BiLSTM model to detect sleep apnea based on EEG and reached a high accuracy on different publicly available datasets [36]. Chang et al. proposed a depression assessment framework based on the spatiotemporal network of EEG and BiLSTM and achieved more than 70% accuracy in the SEED dataset [37].

Two emotional models widely used in the existing research are the circumplex model of affects (CMA) and the discrete emotion model (DEM). CMA defines emotions in a two-dimensional space with arousal and valence. In the work of Posner et al. about CMA, the authors presented that emotion states occur from the cognitive interpretations of core neural sensations and that CMA is a useful tool to study the development of emotion disorders and cognitive underpinnings of affective processing [38]. DEM conversely supposes each discrete emotional state is a different state. In the study of Kılıç et al., the authors proposed that assigning each emotion as a separate discrete state based on DEM is important in recognizing different emotional states and particularly in neuropsychiatric diseases [39].

Motivated by the fact that discrete emotions are vital for emotion recognition and that different entropy features represent implicit EEG complexity in different degrees, we focus on finding out which entropy feature is the best for characterizing three discrete emotional states (i.e., positive, neutral, and negative) and whether integrating different entropy features can enhance the performance of emotion classification by utilizing BiLSTM in the present study. In this paper, we propose a novel framework for cross-subject emotion recognition based on fused entropy features of EEG and BiLSTM. Our approach is to model a BiLSTM classifier based on the fusion of entropy features in EEG induced by different emotional film clips. We first calculate the MSE feature and four other entropy features of EEG to mine the dynamic changes of EEG in different emotion statuses. Then, a BiLSTM classifier is trained to learn the bidirectional time dependency in the extracted EEG features and to realize emotion recognition.

This paper is organized as follows. Section 2 addresses the EEG dataset used in our work. Section 3 details the adopted methodologies. The results of the research are presented in Section 4 and discussed in Section 5. Finally, Section 6 concludes the paper.

## 2. Data Resource

The EEG data in this study came from the 2020 World Robot Competition—BCI Control Brain Robot Contest. It consisted of two public datasets, namely SEED [11] and SEED-FRA [40] (the SJTU Emotion EEG Dataset, https://bcmi.sjtu.edu.cn/home/seed/, accessed on 30 October 2014), collections for various emotion research purposes using EEG provided by Shanghai Jiao Tong University. Prior to the data collection, the experiment was approved by the Ethics Committee, Shanghai Jiao Tong University. The data were gathered from 23 healthy subjects (15 Chinese and 8 French) while they watched different emotional film clips in their native language. First, 50 cinema managers were asked to fill in a questionnaire in which they were supposed to describe the emotional valence of at least three film excerpts for each emotion state (i.e., positive, neutral, and negative). The cinema managers were selected because they were likely to have significant knowledge about films, which might contribute to creating a large preliminary list of film scenes [41]. Then, the listed emotional film excerpts were discussed and viewed by the cinema managers to rate their valence scale from 1 (sad) to 9 (happy) using the Self-Assessment Manikin (SAM). The mean and standard values of each film excerpt were calculated to analyze the rating results, and the initial pool of film clips was established. After this step, a pilot trial was executed to test whether the selected film clips could elicit the expected emotions. According to the SAM rating results of subjects, the mean and standard values of each film clip in the pilot trial could be obtained. Emotional film excerpts from five Chinese films and seven French films with the largest mean values and similar standard values were finally selected as the positive stimuli. There were also twelve film excerpts with the smallest mean values and approximative standard values chosen to be negative stimuli. As for the neutral stimuli, they consisted of film excerpts whose mean values were close to five (the mean value of the valence scale), and standard values were similar. Before the experiment, subjects were asked to finish the Eysenck Personality Questionnaire (EPQ), and only those with stable moods were selected. There are three types of emotions (i.e., positive, neutral, and negative) included in the experiment, and each type of emotion had five (for Chinese subjects) or seven (for French subjects) corresponding film clips. Each emotional film clip lasted for 2 min.

**Figure 1:**
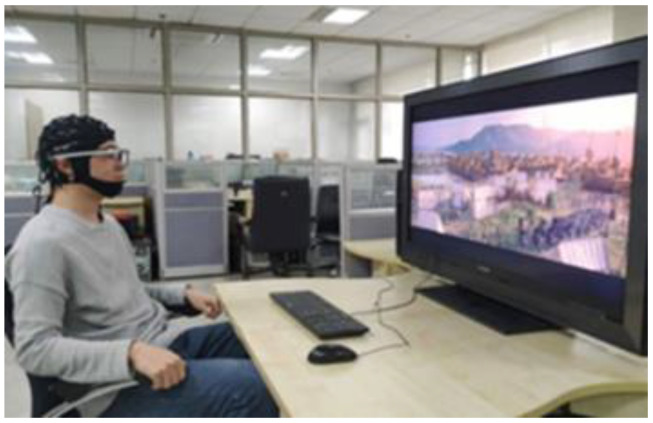
The emotion experiment scene.

**Figure 2:**
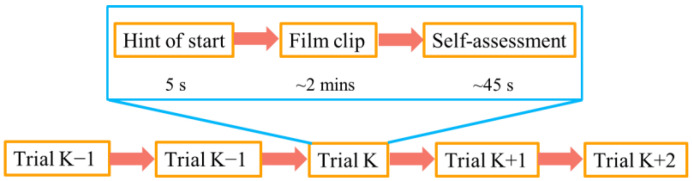
Protocol of the experiment.

The experiment was performed in a quiet room. Figure 1 shows the experiment scene. A 62-channel electrode cap arranged according to the international 10–20 system was used to collect the EEG data. The sampling rate was set to 1000 Hz. Before starting, all subjects were given written and oral instructions on the experiment and were asked to stay as still as possible and refrain from moving. In the experiment, the subjects sat comfortably and paid attention to watching the forthcoming film clips. Eight of the subjects watched 21 film clips (i.e., 21 trials), with seven film clips corresponding to each emotion. The other fifteen subjects were shown 15 film clips, and there were five corresponding film clips for each emotion. The detailed protocol of the experiment is shown in Figure 2. A 5 s picture hint was set before each clip, and there was a 45 s interval after each clip, allowing the subjects to report their emotional states concerning the film clips based on their feelings. The self-reported emotional states were then used to validate the emotion classification results of the study. The details about the database can be found in [11,37,40].

## 3. Methodology

The analysis process of EEG-based emotion recognition includes three steps: data preprocessing, feature extraction, and emotion recognition. This section describes how we dealt with the data in detail. The analyzing process was implemented in MATLAB 2019b.

### 3.1. Preprocessing

As previous studies have demonstrated that some electrodes are irrelevant to emotion changes [42], and Zheng et al. found that the lateral temporal brain area is activated more than other brain areas in emotion processing [11], we first selected twelve electrodes (i.e., FT7, T7, TP7, P7, C5, CP5, FT8, T8, TP8, P8, C6, CP6) in the lateral temporal brain area for further research in this paper. The EEG data were then down-sampled to 256 Hz to improve the calculation efficiency. After that, the EEG segments corresponding to each film clip’s duration were extracted to obtain the entire EEG data from watching all the film clips, as the raw EEG data contained the EEG signals not only while watching the films but also in the preparation and self-assessment stages. To reject interference from the power line, we used a bandstop filter of 50 Hz. Five frequency bands (i.e., delta, theta, alpha, beta, and gamma) of the EEG signals were then roughly extracted, applying wavelet decomposition. Finally, a wavelet-based technique was used to remove the artifacts in each band.

Wavelet transform, which is an effective time-frequency analysis method with the ability for good local representation of signals in the time and frequency domain, is usually used to analyze EEG signals [43]. By decomposing the signal at each level, the detailed and approximate component wavelet coefficients can be obtained corresponding to the level. The wavelet coefficients could reflect the detailed information of the signal as well as the correlation with the mother wavelet. In fact, the coefficients of the artifacts are usually larger than those of a normal EEG signal. Therefore, artifacts were eliminated by setting a threshold value [44]. This wavelet-based method has been validated to be effective in the field of driver fatigue assessment [45,46]. We can calculate the threshold by

(1) $$T_{j}=mean\left(C_{j}\right)+2\times std(C_{j})$$

where *Cj* is the wavelet coefficient at the *j*th level of wavelet decomposition. The value of any coefficient is larger than *Tj*; it is considered a coefficient of the artifact and halved to eliminate its influence. Then, the wavelet-corrected signal can be reconstructed with the new set of wavelet coefficients. More details about the preprocessing process can be found in Appendix A.

**Figure 3:**
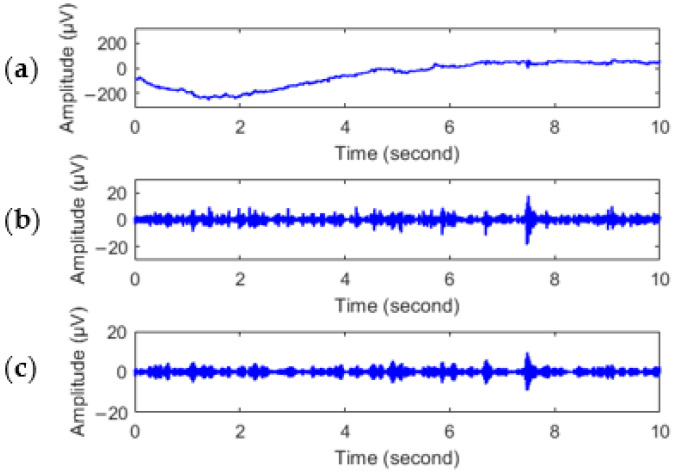
EEG signal preprocessing; the units are µV. (**a**) The original EEG signal; (**b**) the gamma wave obtained by wavelet decomposition; (**c**) the gamma wave after artifact removal.

As usually used to resemble EEG signals in the literature [47], db6 was selected as the mother wavelet. The EEG signals from all the twelve electrodes were preprocessed in the same way as mentioned above in this paper. Figure 3 shows the preprocessing results of the gamma frequency band at the FT8 electrode. Figure 3a is a 10 s duration of the original EEG signal with various artifacts. Figure 3b gives the gamma frequency band extracted by wavelet decomposition. The body movements caused large fluctuations in the gamma band were obviously removed, though artifacts induced by blinks still exist. The result of the artifact removal is shown in Figure 3c. It is clear that the wavelet-based thresholding technique can reduce the interference of artifacts in Figure 3b.

### 3.2. Feature Extraction

Five entropy features were calculated to explore the dynamic changes in EEG induced by different emotional film clips including MSE, AE, FE, DE, and Rényi entropy (RE).

#### 3.2.1. Multi-Scale Entropy

MSE, with the ability to reduce the interfere of residual noise on the results by calculating features in different time scales, was chosen to manifest the dynamic changes while subjects were viewing emotional films [48]. It was proposed firstly by Coasta et al. in 2003 [49] that MSE could reflect the complexity of signals in different scale factors by extending the idea of SE to several time scales. For the EEG signal {*x_1_*, …, *x_i_*, …, *x_N_*}, it is first coarse-grained according to a specified scaling factor *τ*. In this process, the original signal is divided by sliding windows with a length of *τ*, and the average value is then calculated in each window to obtain the coarse-grained time series {*y*^(*τ*)^}. It is defined as

(2) $$y_{j}^{\left(\tau \right)}=\frac{1}{\tau }\sum_{i=\left(j-1\right)\tau +1}^{j\tau }x_{i},\;1\leq j\leq \frac{N}{\tau },$$

Then, the SE of the simplified time series {*y*^(*τ*)^} is calculated at each time scale. For more information about the calculation of SE and the parameter setting, please see Appendix B.

#### 3.2.2. Approximate Entropy

AE proposed by Pincus in 1991 is a kind of nonlinear dynamics parameter to measure the complexity and the statistical quantization characteristics of the signal [50]. Due to its effectiveness in reflecting the structure characteristics and complexity information of signals with fewer data points, it is widely used in time series classification studies. Its formula is

(3) $$\mathrm{AE}\left(m,r,N\right)=\frac{1}{N-m+1}\sum_{i=1}^{N-m+1}\log C_{i}^{m}\left(r\right)-\frac{1}{N-m}\sum_{i=1}^{N-m}\log C_{i}^{m+1}\left(r\right),$$

where $C_{i}^{m}\left(r\right)$ can be calculated by

(4) $$C_{i}^{m}\left(r\right)=\frac{B_{i}^{m}}{N-m+1},$$

where $B_{i}^{m}$ is the number of matches of dimension *m*.

The mode dimension *m* is set as 2, and the tolerance *r* is equal to the standard deviation of the signal times 0.2. *N* is the data length, which is set as 256, equals the data points in a 1 s time window without overlap.

#### 3.2.3. Fuzzy Entropy

FE is also a measure of the complexity of signals like AE. Instead of the Heaviside Step Function used in AE, the concept of the fuzzy set is introduced into FE to measure the similarity of two vectors. An exponential function is chosen as the fuzzy function that enables the FE values to change smoothly and continuously with parameters change [51]. In this case, FE is also calculated in our study to make a comparison with other entropy features. It can be calculated by

(5) $$\mathrm{FE}\left(m,n,r,N\right)=\ln\frac{O^{m}\left(n,r\right)}{O^{m+1}\left(n,r\right)},$$

where *O^m^*(*n*,*r*) is the mean value of the fuzzy membership of the time series with length *N* in dimension *m* and tolerance *r*; *n* is used to determine the gradient of the similarity tolerance boundary. More details about the definition of *O^m^*(*n*,*r*) and the parameters in Equation (5) are described in detail in Appendix B.

#### 3.2.4. Rényi Entropy

RE is a generalization of Shannon entropy, which reflects the time-frequency features and randomness of signals. It is widely used in information theory, such as classification problems. For a given EEG signal *X* = {*x*_1_, …, *x_i_*, …, *x_N_*}, its RE can be calculated by

(6) $$\mathrm{RE}=\frac{1}{1-q}\log\left(\sum_{i=1}^{N}p{\left(i\right)}^{q}\right),\;\;q\geq 0\;\&\;q\neq 1,$$

where *q* is the entropic index, *p*(*i*) is the probability of choosing *x_i_* in *X,* and $\sum_{i=1}^{N}p\left(i\right)=1$.

According to Kar et al.’s [52] study, we use *q* = 2 to calculate the two-order entropy in a sliding window with a length of one second.

#### 3.2.5. Differential Entropy

As an extension of Shannon entropy, DE can be utilized to reveal the complexity of a time series [53]. Previous study has proven that DE performs better than energy spectrum and asymmetrical features in EEG-based emotion state detection [54]; thus, we calculated DE to represent the changes in EEG signals in different emotional films. It is defined as

(7) $$\mathrm{DE}=-\int_{a}^{b}f\left(x\right)\log\left(f\left(x\right)\right)dx,$$

where *f*(*x*) is the probability density function of the time series and [*a*, *b*] is the taking value interval. If the time series is approximately a Gaussian distribution *N*(*μ*,*σ*^2^), its DE can then be calculated by

(8) $$\mathrm{DE}=-\int_{-\infty }^{+\infty }\frac{1}{\sqrt{2\pi \sigma^{2}}}e^{\frac{{\left(x-\mu \right)}^{2}}{2\sigma^{2}}}\log\left(\frac{1}{\sqrt{2\pi \sigma^{2}}}e^{\frac{{\left(x-\mu \right)}^{2}}{2\sigma^{2}}}\right)dx=\frac{1}{2}\log2\pi e\sigma^{2},$$

where *μ* and *σ*^2^ are the expectation and variance of the time series. In our present work, DE is extracted from the signals in a sliding window of a length 1 s without overlap.

### 3.3. BiLSTM

**Figure 4:**
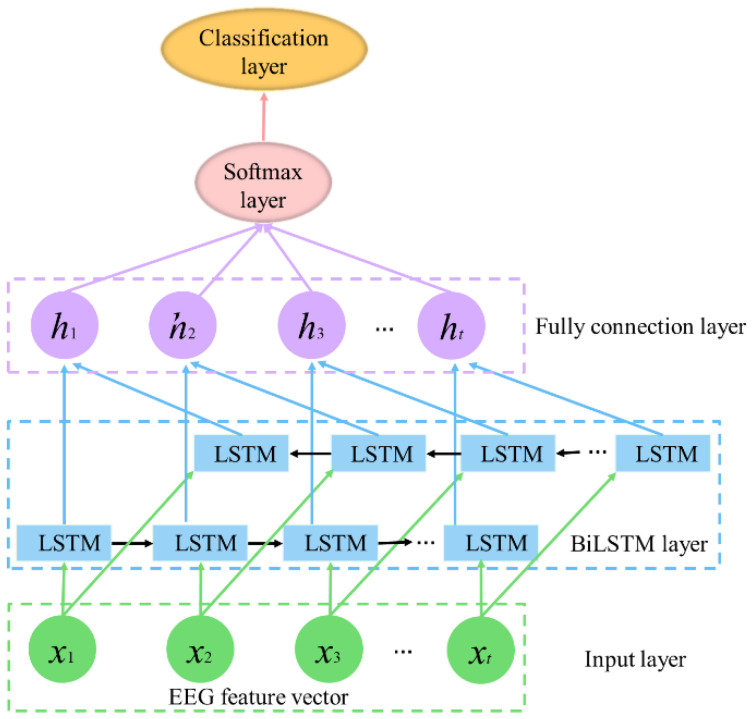
BiLSTM network architecture. It consists of five layers (input layer, BiLSTM layer, fully connection layer, softmax layer, and classification layer). *x_t_* is the EEG feature of time *t*. *h_t_* is the hidden state of LSTM cell in time *t*.

As an update to LSTM, BiLSTM not only possesses the ability to avoid the receding gradient problem but also memorizes long- and short-term dependency of EEG in a forward direction as well as in a backward direction [55]. As shown in Figure 4, we can see that BiLSTM works in a way that integrates two LSTM together composed of LSTM memory cells.

**Figure 5:**
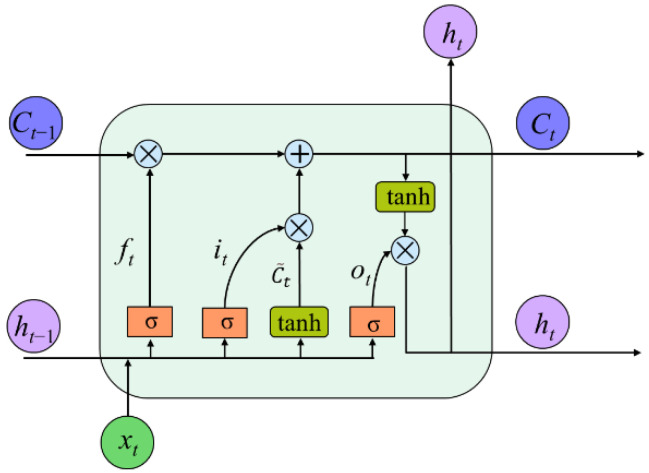
The details of a LSTM memory cell. It contains two kinds of activation functions (σ and tanh). *C_t_* is the LSTM cell state in time *t*. *f_t_*, *i_t,_* and *o_t_* represent the outputs of forget gate, input gate, and output gate in time *t* separately.

LSTM memory cells contain four neural network layers compared to conventional RNN cells with only one layer to model the long-term context. The structures called “gate” consist of neural network layers, and their interactions make it possible for a LSTM memory cell to add or remove information from the cell state [56]. The details about how the memory cell works can be found in Appendix C. Figure 5 shows the structure of a LSTM memory cell.

## 4. Results

### 4.1. Feature Extraction

**Figure 6:**
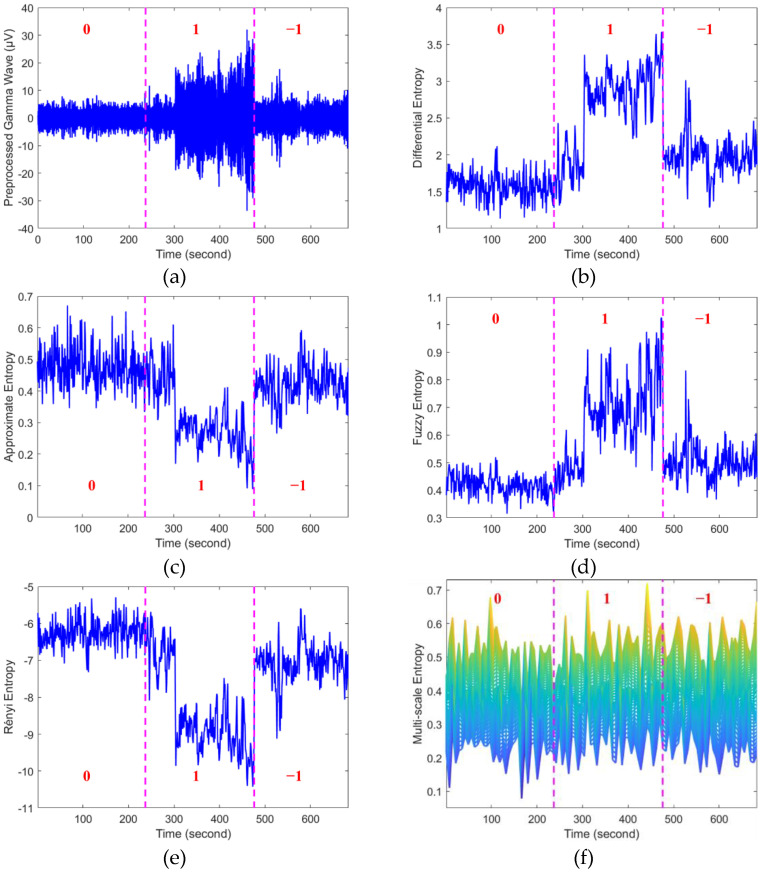
The results of five entropy-based features of EEG. The numbers “1”, “0”, and “−1” represent positive, neutral, and negative emotions, respectively. The purple dashed lines show the boundaries of different film clips. (**a**) Preprocessed gamma frequency band; (**b**) DE; (**c**) AE; (**d**) FE; (**e**) RE; (**f**) MSE.

In this paper, the five-scale MSE feature was first extracted from the five frequency bands for the selected twelve electrodes after preprocessing. Four other kinds of entropy-based features mentioned in Section 3.2 were also calculated. A non-overlapped sliding window of 1 s was used in the feature extraction procedure. The dimension of the obtained MSE feature matrix for each subject is 300 × *N*, and the other features (i.e., AE, FE, RE, and DE) share the same dimension as 60 × *N*, where *N* stands for the sampling time. Figure 6 shows parts of the preprocessed gamma frequency band and its entropy features in FT8. The red numbers in Figure 6 are the emotion types of the film clips’ duration, which are in accordance with the self-reported emotional states. A positive emotion is marked as “1”, and “0” and “−1” represent neutral and negative emotions, respectively. The interval between two adjacent purple dashed lines corresponds to the film clip.

It can be seen from Figure 6 that the waveform of the gamma band after preprocessing varies in different emotional films, and the five entropy-based features change regularly corresponding to the film clips. In Figure 6a, the amplitudes of watching a positive film are obviously larger than those of the other two emotional films. The amplitudes of negative movies rank in second place, followed by those of neutral movies. The fluctuations of DE and FE are similar, as shown in Figure 6b,d. The highest peak values occur during positive emotion film clips, and the lowest valleys appear while subjects watch neutral film clips. The feature values of watching negative film clips are positioned between these two conditions. Additionally, AE and RE share the same waveforms, as can be seen in Figure 6c,e, which are contrary to those of DE and FE. As for the result of MSE in Figure 6f, there is a slightly increasing tendency in the values of MSE when subjects were watching positive and negative films compared with the neutral films, whereas no obvious differences can be seen between positive and negative films in MSE.

### 4.2. The Classification Results of BiLSTM

BiLSTM is applied to classify the emotion states of the subjects in order to explore the long-term dependency and interplay of the extracted features at different times. We trained BiLSTM models for each kind of feature and the fused entropy features separately. Then the performance of BiLSTM utilizing a single-type feature was compared with that of fused entropy features. Further, the result was also compared with conventional LSTM to make the results more convincing. The five types of feature matrixes obtained in Section 4.1 were first normalized to (−1, 1) to eliminate the effect of individual differences. Then, the normalized feature matrixes could be directly fed into the classifier. As for the output (i.e., the category label vectors), it can be set according to the sequence of the film clips and the results of the self-assessment. The dimension of the label vectors is 1 × *N*, where *N* is the sampling time. There are in total three categories in this paper: positive, neutral, and negative. The training data come from eighteen subjects selected randomly from all, and the data of the remaining five subjects was set as testing data. The recognition accuracy was defined as the average accuracy of the five subjects in the testing group. The results of different entropy features are shown in Table 1. “ALL” means the five entropy features.

From Table 1, it can be seen that the performance of BiLSTM is clearly better than that of LSTM. As for the result comparison the two models based on single-feature inputs, LSTM and BiLSTM with the input of MSE achieves the best result, reaching at 66.12% and 67.9%, respectively, and they are slightly higher than those of DE, which was proven to be a better feature to classify different emotion states in a previous study [11]. The RE feature leads to the lowest accuracy of 54.23% in LSTM and 57.15% in BiLSTM, and the accuracies of AE and FE lie between RE and AE. Furthermore, the two models’ performance is apparently enhanced while using fused entropy features to detect emotion states peaking at 67.22% in LSTM and 70.05% in BiLSTM.

## 5. Discussion

Different types of entropy features of EEG were calculated to explore the dynamic changes of EEG while subjects were watching emotional film clips, and the results of the gamma frequency band in FT8 are shown in Figure 6. It can be seen that the entropy values are significantly different for different emotional films. The higher peaks appear in positive and negative films in the results of DE, FE, and MSE, and the lowest valleys occur in neutral films. Subjects are highly stimulated in emotion when exposed to the positive and negative films; in these cases, the brain activity is usually active and complex (see Figure 6a), and it objectifies a high complexity in the gamma band. However, subjects are not as immersed in the neutral films as during the other two conditions. Thus, there is a decrease in complexity and the lowest entropy values appear. The results are accordant with previous studies showing that greater activities of the gamma band can be found in positive states and the lowest activities are in neutral conditions [15,57]. As for the results of RE, the positive films induce the largest absolute values followed by negative films, and the smallest absolute values are caused by neutral films. The results are consistent among DE, FE, and MSE. RE is a reflection of the amplitude’s distribution; a smaller RE is obtained when the EEG amplitude concentrates in a certain subsequence, indicating the signal is more ordered and less complex [58]. Hence, a larger absolute value of RE can be seen in a more active gamma band when subjects are greatly affected by positive and negative films. Subjects are usually more engaged in viewing positive film clips than neutral clips, generating more active brain activities, but the complexity changes in EEG may be not large enough to be detected by AE. Different from the changing rules of the above four features, AE reaches its highest value in neutral emotion and its lowest value in positive emotion, as shown in Figure 6c. As we know, AE was proposed to measure the average logarithmic conditional probability of the new pattern’s occurrence in a signal with the dimension change [50]. It introduces self-matches into calculation and will inevitably lead to calculation bias, which can result in an insensitivity to small changes in complexity [59]. Thus, the small complexity changes in brain activity during the positive film’s duration may be ignored. It decreases the detected new patterns and leads to the lower AE value in positive emotion.

The extracted entropy features are used to detect emotion states by training a BiLSTM model. The classification results in Table 1 illustrate that the mean accuracy of MSE is slightly higher than that of the other four entropy features using BiLSTM, as it can reveal the complex information of EEG in film watching as well as reduce the influence of the residual noise on the results to some extent [60]. Moreover, the performance of BiLSTM is further improved with multiple entropy features, reaching to 70.05%. Different features can compensate for each other according to the study in [23]. Then, the more useful information of EEG features can be learned by BiLSTM, thus contributing to a higher accuracy in emotion recognition. Additionally, the traditional one-directional LSTM was also trained to make a comparison with BiLSTM. Table 1 indicates that the trained BiLSTM performs better than LSTM since it can learn the long- and short-term dependency among EEG features in a forward direction and in a reverse direction [55]. This finding is consistent with the finding that utilizing the BiLSTM classifier is an efficient way to decrease the train and test error and to increase the classification accuracy [61]. The accuracy is comparable with that in the research in [26], who utilized the DE feature and transfer learning to detect three emotions and reached an accuracy of 72.47% in the SEED dataset. However, our accuracy is lower than that in [11] who used the DE feature and DBN to recognize different emotions in the SEED dataset. This might be because SEED includes only Chinese subjects, and the data we obtained from the competition include not only Chinese but also French subjects (i.e., the data used in our study consist of two datasets: SEED and SEED-FRA). Although the subjects watched their native language films during the experiment to elicit emotional changes more easily, and the stimulus types of the films are the same, there are differences between the Chinese and French subjects, which may lead to the lower accuracy in our study. In a study utilizing the same data as ours, a similar accuracy of about 70% was obtained in [37] for depression recognition.

This study shows the feasibility of recognizing emotion status by deploying multiple entropy features. However, as the dataset we used contains subjects and stimuli of two native languages, and film clips in different emotional categories vary in the degree of induced emotion, there are still limitations in the present work. First, the used dataset consisted of two public datasets, which involved both Chinese and French subjects and stimuli. Each subject watched films in his native language to elicit emotional changes more easily, but the number of film clips for the two languages was different. Since the stimulus types of the films were the same, we assume the different number of film clips for the subjects had no effect on the results in the present work, though influences we do not realize might exist in the results because of the different native speakers and the number of film clips. Further, subjective labeling of participant emotional states was adopted to recognize the emotions in our study, as it is beneficial to the feature analysis for the same type of emotion states and to ensure the reliability of the results by providing more accurate feelings of the subjects. Although most of studies about emotions in the literature choose subjective labeling, there is research that selects objective labeling to label the emotional states, which can be further studied in our future experiment design. Additionally, the results obtained in our present work can be further improved by utilizing some new, more effective algorithms. Electrical Source Imaging (ESI), an emerging algorithm to reconstruct brain or cardiac electrical activity from electrical potentials measured away from the brain, can determine the location of current sources from measurements of voltages [62]. This would be a novel and interesting topic for estimating the cortex brain regions involved in each video viewing to improve the emotion recognition accuracy in our future work.

## 6. Conclusions

In this paper, we proposed a cross-subject emotion recognition framework based on fused EEG entropy features and a BiLSTM classifier. It demonstrates that MSE is more effective in analyzing the complex emotion information in EEG than the other entropy features when adopting a single EEG feature. What is more, the classification accuracy can be apparently increased by combining all entropy features, which proves that there is information compensation among different types of features.

Future work mainly includes two aspects. One aspect is to extract more features from different perspectives, such as time-frequency domain features and non-linear dynamic features, for feature fusion; the other is enhancing the performance of the classifier with a new feature fusion algorithm or by estimating the cortex brain regions involved in watching emotional film clips by applying Electrical Source Imaging.

## Figures and Tables

**Table 1:** The mean accuracies of BiLSTM and LSTM for different features (%).

Feature	AE	FE	RE	DE	MSE	ALL
LSTM	61.1	59.47	54.23	65.09	66.12	67.22
BiLSTM	63.43	61.1	57.15	66.34	67.9	70.05

## Data Availability

Publicly available datasets were analyzed in this study. This data can be found here: [https://bcmi.sjtu.edu.cn/home/seed/, accessed on 30 October 2014].

## Appendix A. Preprocessing

In this section, we preprocessed the EEG signals to remove the artifacts and obtain five frequency bands. We first downsampled the signals from 1000 Hz to 256 Hz to enhance the calculation efficiency. Then, the EEG segments during film clip watching were extracted. After that, to obtain different frequency band rhythms, a bandstop Butterworth filter of order 4 with cutoff frequencies of 0.19 and 0.2 was used to remove the power line, and wavelet decomposition was applied to roughly obtain the five frequency bands of EEG signals. A wavelet-based technique was chosen to remove the artifacts in the end.

Wavelet transform is a well-known time-frequency analysis algorithm and has been widely adopted to deal with non-stationary signals. The original signals are decomposed and expressed with a scaled and shifted version of the mother wavelet *ψ*(*t*) and a scaling function *ϕ*(*t*) [52]. The discrete mother wavelet can be expressed as

(A1) $$\psi_{j,k}\left(t\right)=2^{\frac{j}{2}}\psi \left(2^{-j}t-k\right),\;\;\;\;\;\;\;j,k\in Z,$$

The signal *S*(*t*) can then be represented as

(A2) $$S\left(t\right)=\sum_{k}s_{j}\left(k\right)\phi_{j,k}\left(t\right)+\sum_{k}d_{j}\left(k\right)\psi_{j,k}\left(t\right),$$

where *s_j_*(*k*) is the approximate coefficient at the *j*th level, and *d_j_*(*k*) represents the detailed coefficient.

In this study, the EEG signal was decomposed into seven levels using db6 (Daubechies family), as the waveform of db6 is similar to the EEG signal and it has been widely used in decomposing EEG in the literature [47]. The delta (1–4 Hz), theta (4–8 Hz), alpha (8–13 Hz), beta (13–30 Hz), and gamma (30–50 Hz) frequency bands were roughly obtained by reconstructing the detailed components at levels of seven, five, four, three, and two, respectively.

To eliminate the artifacts’ interference, the five frequency band rhythms are decomposed separately into three levels with a 1 s sliding window, and the thresholds of each level are calculated by Equation (1). The coefficient is halved if its value is larger than the calculated threshold. In this way, a new set of signals is generated without artifacts.

## Appendix B. Feature Extraction

This section lists some details about the well-known formulas and the parameter setting to extract the entropy features of the five frequency bands, which provides supplementary information for Section 3.2.

After the first coarse graining step in extracting the MSE feature (see Section 3.2.1), the SE is then calculated for the new time series in different scales by the following formula

(A3) $$\mathrm{SE}\left(m,r,N\right)=-\ln\left[\frac{B^{m+1}\left(r\right)}{B^{m}\left(r\right)}\right],$$

where *m* is the mode dimension of the data vector, *r* is the tolerance for similarity matches, and *N* is the number of data points. $B^{m}\left(r\right)$ and $B^{m+1}\left(r\right)$ are the numbers of matches for dimension *m* and *m* + 1, respectively.

In this paper, the five-scale of the MSE feature is calculated with a 1 s sliding window for the five frequency bands. The mode dimension *m* is set as 2, and the tolerance *r* is equal to the standard deviation of the signal times 0.2.

As for FE in Section 3.2.3, the parameter *O^m^*(*n*,*r*) in Equation (5) is defined as

(A4) $$O^{m}\left(n,r\right)=\frac{1}{N-m}\sum_{i=1}^{N-m}\left(\frac{1}{N-m-1}\sum_{j=1,j\neq i}^{N-m}D_{ij}^{m}\right),$$

(A5) $$D_{ij}^{m}=f\left(d_{ij}^{m},n,r\right),$$

(A6) $$X^{m}\left(i\right)=\left[x\left(i\right),x\left(i+1\right),\;\ldots ,x\left(i+m-1\right)\right],$$

(A7) $$X^{m}\left(j\right)=\left[x\left(j\right),x\left(j+1\right),\;\ldots ,x\left(j+m-1\right)\right],$$

where *n* is the fuzzy exponent, and $\;d_{ij}^{m}$, the distance between vectors *X^m^*(*i*) and *X^m^*(*j*), is defined as the absolute value of the maximum difference between the corresponding elements in the two vectors. $D_{ij}^{m}$ is then calculated by the fuzzy function *f*($d_{ij}^{m}$,*n*,*r*) to show the similarity of vectors *X^m^*(*i*) and *X^m^*(*j*).

The same parameters of *m*, *r,* and *N* with AE are used. *n* is set as 2 according to the research of Chen et al. [51].

## Appendix C. BiLSTM Classifier

As mentioned in Section 3.3, BiLSTM is an integration of two opposite-direction LSTM and consists of LSTM memory cells. A LSTM cell contains an input gate, output gate, and a forget gate (see Figure 5), which serve to protect and control the cell state *C_t_*. The first step in a LSTM cell is to select that which should be deleted in *C_t_*_−1_, and it is realized by the forget gate:

(A8) $$f_{t}=\sigma \left(W_{f}\cdot \left[h_{t-1},x_{t}\right]+b_{f}\right),$$

Then, the input gate consisting of a sigmoid layer chooses what kind of values need to be updated (Equation (A9)), and a tanh layer creates a new candidate cell state ${\tilde{C}}_{t}$ from Equation (A10). Following these two steps, the new cell state *C_t_* can be obtained from Equation (A11):

(A9) $$i_{t}=\sigma \left(W_{i}\cdot \left[h_{t-1},x_{t}\right]+b_{i}\right),$$

(A10) $${\tilde{C}}_{t}=\tanh\left(W_{C}\cdot \left[h_{t-1},x_{t}\right]+b_{C}\right)$$

(A11) $$C_{t}=f_{t}*C_{t-1}+i_{t}*{\tilde{C}}_{t},$$

In the end, the output gate and a tanh layer are activated to decide the final output *h_t,_* as expressed in Equations (A12) and (A13):

(A12) $$o_{t}=\sigma \left(W_{o}\cdot \left[h_{t-1},x_{t}\right]+b_{o}\right),$$

(A13) $$h_{t}=o_{t}*\tanh(C_{t})$$

In the equations, *σ* and tanh represent the activation functions, *x_t_* denotes the input of the cell (the EEG feature of time *t*), and *W*, *b* and *h* are the weight, bias, and hidden state of the gates, separately.

In this paper, we trained and tested various BiLSTM and LSTM classifiers with different parameters to find the best parameters for each feature. The Adam optimizer was utilized as the optimizer of the classifier. We selected the data of 18 subjects as the training set, and that of the remaining five subjects was the testing set. The average accuracy of the test set was used to evaluate the classifier’s performance. The details of the parameters tested are listed in Table A1.

**Table A1:** The detailed parameters trained in BiLSTM and LSTM.

Name	Value
Hidden units	[10:150] with step of 10
Epochs	[50:150] with step of 20
Mini batch size	[50:100] with step of 10
Learning rate	0.001
